# 1904

**Published:** 1904-12-31

**Authors:** 


### Hospital Clinics.

### 1904.

The year that is now closing has been a period of healthy activity in every department of medicine, and although so far as we are aware it has not seen the birth of any epoch-making discovery, yet it may be regarded with satisfaction in the light of the sterling work which has been done—a sentiment which need not be associated with any prospect of future repose, but will be accompanied, rather, by a hope of greater efforts and achievements to come.

### State Medicine.

The prevention of ill-health and disease may properly be said to be the ultimate aim of all medical endeavour, but the application of the principle to mankind collectively has never yet received the encouragement or consideration it deserves. The treatment of individuals has been glorified out of proportion to its achievements, while State medicine has been compelled to act the part of a poor and neglected Cinderella. The public are apathetic towards the subject; and yet of all the callings open to the higher intellects of men, that which is directed towards preserving the health of the community is one of the greatest, whether judged from a financial or from a humanitarian point of view, and the emoluments and honours offered for the service might well be made commensurate with the issues involved. Nevertheless, there are indications that the public are beginning to appreciate the importance of preventive medicine in a few of its branches. The daily Press has shown an increasing readiness to devote some of the space in their news columns to these and similar matters. In this way a good deal of attention has been given to many medical subjects. Tropical medicine, the prevention of tuberculosis and its hygienic treatment are cases in point. Physical degeneration and, as part of the same problem, the proper feeding and hygiene of children have been prominently before the public. The disgusting condition of our milk supply and the evils for which it is responsible have been impressively represented to the public, and there are some signs that the heavy toll of infant life which is paid to our present slovenly methods is likely to produce a reformation of our arragements for milk collection and distribution.

We have placed this growing appreciation of general health problems on the part of the public in the first place, both because we believe it to be fraught with the greatest promise for the future, and because the public Press of to-day offers a field which is still sadly in need of medical cultivation. When the public is better educated, great advances will be possible, and much preventable suffering and loss of life will be prevented. But so long as the discussion of public health questions remains limited to technical papers and professional societies, so long will the hygienic millennium be postponed.

### Tropical Medicine.

There has been a steadily increasing energy in all departments of tropical medicine. So far as research is concerned, perhaps trypanosomiasis and beri-beri have been the most important objects. In the former careful observations have been made with a view to finding some drug that will play the same part in trypanosomiasis that quinine does in malaria. Some success appears to have followed the use of arsenic and trypan red, but further experiments are yet necessary before a definite opinion can be expressed with regard to the therapeutic value of these drugs. Laveran is at work on the preparation of a serum which may prove to be of some value.

Several statistical reports have appeared during the year showing the very great success that has followed upon rational measures of preventing malaria by organising mosquito campaigns and taking other precautions against infection by mosquitos. A natural outcome of all this progress has been the establishment of diplomas of tropical medicine. The University of Cambridge took the lead in instituting a diploma in tropical medicine and hygiene, and shortly afterwards the Liverpool University followed their example. At the present time the Royal College of Physicians, England, and the Royal College of Surgeons, London, are jointly considering the question of holding an examination for a diploma in tropical medicine. So that in the future candidates will have ample opportunities for obtaining the encouragement of a satisfactory technical credential.

### Tuberculosis.

In the course of the year there appeared a most important document in the form of an interim report issued by the Royal Commission appointed in 1901 to inquire into the relations of human and animal tuberculosis. This report set forth authoritatively certain facts relative to the transmissibility of human tuberculosis to animals, which must serve in the future to guide the medical profession, and perhaps also the Legislature, as to the correct attitude to be adopted towards this subject when any steps for the prevention of tuberculosis are under consideration. For the purposes of the inquiry more than 200 bovine animals were experimented with, and over 20 strains of tuberculous material of human origin were employed. The results obtained with these were compared with the effects produced by several different strains of tuberculous material of bovine origin. Not one of the strains failed to produce tuberculosis in animals subjected to inoculation, and in some instances the resulting disease ran an acute course.

In conclusion, the Commission stated that, after carefully comparing in every detail the disease thus set up in the bovine animal by material of human origin with that set up in cattle by material of bovine origin, they have been unable to discover any character by which the one could be distinguished from the other. Hence, until the contrary is proved, which seems extremely improbable, it must be assumed that bovine tuberculosis is a source of danger to the human being; and Koch's view of the distinction between human and bovine tuberculosis must for the present be laid aside.

Material results are also likely to be the outcome of an inquiry which was carried out by Mr. Martin, Dr. Haldane, and Mr. Arthur Thomas into the causes of the excessive mortality of Cornish miners, and the means by which such excessive mortality might be avoided. These gentlemen, it will be remembered, soon discovered that ankylostomiasis was prevalent among the miners in certain districts in Cornwall, and they were able to offer, in a report made to the Secretary of State, suggestions as to the preventive measures which might be taken. In the course of the past year they made a second report dealing with miner's phthisis. They attribute the excessive mortality from this cause to the inhalation of dust raised by machine rock-drills. They further state that a completely effective remedy for this is afforded by a spray of water passing into the holes; and they suggest also that ore should be damped before it is moved with the same object of preventing the inspiration of irritating dust. A similar inquiry which resulted in similar conclusions was carried out almost simultaneously in South Africa.

With regard to the treatment of tuberculosis there has been a wide expansion of sanatorium accommodation, and this is all to the good. But no finality has been reached; the demand is still a long way in advance of the supply, and sanatoria are especially needed for cases of so-called surgical tuberculosis.

### Cancer.

Though a great deal of energy has been spent upon cancer research no great result has hitherto been attained. Much, no doubt, has been done towards clearing the field of erroneous hypotheses, and a large number of facts has been collected which may pave the way to future discovery. But beyond this there is little room for contentment. The chief facts which have been ascertained or placed upon a more certain footing are the liability of all classes of the vertebrate kingdom to malignant growth, the intransmissibility of cancer from individuals of one species to those of another, and the peculiar nature of the mitotic changes in cancer cells. So far as practical results are concerned, there is but little to say. Investigations are in progress with regard to the possibility of a rational serum diagnosis and serum treatment. The powers of radium as a curative agent have been examined but have yielded no promise of general utility. Greater attention has been directed to precancerous conditions and their operative treatment, and the clamour of surgeons for better opportunities for operating on malignant growths at the earliest possible moment continues with unabated force. This is the theme of a weighty warning to the public contained in the report of the Executive Committee of the Imperial Cancer Research Fund, and it is indeed the chief practical observation which the Executive Committee were able to make; and Mr. Mayo Robson, in delivering the Bradshaw lecture before the Royal College of Surgeons of England chose the occasion for a strong protest "both against the fatalistic tendency to delay and the senseless running after false gods in the treatment of this fell disease." It is to be hoped that the public will pay heed to these words of wisdom.

### Milk Supply.

Renewed attention has been paid to the unsatisfactory condition of our milk supply. It is unquestionable that a multitude of infants die every year because they are fed upon contaminated milk, and in order to combat this evil, sterilised milk depôts are sprouting up in numerous centres of population throughout the country. These depôts have for their main object the supply of sterilised milk for infants, at a cost which will be within the reach of the very poor. But infants are not the only members of the community who are poisoned by milk; it has been conclusively demonstrated that the adult members also frequently suffer severe consequences from the same cause. Therefore, even if sterilised milk depôts were perfect institutions so far as infants are concerned, which they are not, we should still require some protection for the remainder of the community. For this reason, and also because babies do not thrive upon sterilised milk as they do upon fresh milk, we suspect that the milk depôts may in the long run prove to be an evil rather than a blessing to the community; and, further, their wide-spread establishment is likely to result in a postponement of more radical measures. The main difficulty to be surmounted in instituting reform is the cost. It is manifestly undesirable to raise the price paid by the consumer for his milk, even by a little.

A useful comparison has been made between our water supply and our milk supply. It has been pointed our how ineffectual it would be to supply foul water to the population and expect them to boil it before use. But that is the present-day method of dealing with milk. Our water supply costs the community a large annual sum, but a fraction of that sum would probably cover the cost of providing for a reasonable degree of purity of the milk. Healthy cows, clean milking, and the prevention of contamination and decomposition during its distribution are the essential requirements at the present day. It is the growing appreciation of these facts and the increasing desire for some reform that has led us to discuss the matter in a medical review of the year's work.

### A Bacterial Test for Polluted Air.

A method of estimating the degree of pollution of a given sample of air by a quantitative bacterial test has been suggested by Dr. Mervyn Gordon, and it seems to offer such promise for the future that it is well worthy of a prominent place among the scientific achievements of the year. His experiments have a two-fold purport. In the first place, they throw light on the mechanism of personal infection without contact; and, in the second place, they provide a quantitative bacterial test for air comparable to that which has been employed with such great success in estimating the purity of water, and which involves a numerical estimation of the colon bacilli contained in a given sample. Dr. Gordon was able to find among the micro-organisms of the human mouth one, which he describes as streptococcus brevis, which can nearly always be isolated, which is present in large numbers, and which when found in the atmosphere may be regarded as evidence of oral contamination of that atmosphere, just as the presence of bacillus coli may be regarded as evidence of fæcal contamination of a sample of water. Tests showed that the air of a room previously free from the streptococcus brevis, at the end of loud oration by a series of speakers, became contaminated with the characteristic micro-organism even to a distance of 40 feet in front of the speakers, and of 12 feet behind them.

If the value of Dr. Gordon's method is supported by the practical experience of other bacteriologists, it is difficult to conceive a limit to its useful application. The customary quantitative estimation of the carbon dioxide in the air has long been known to be a very indifferent guide to the fitness of the air for respiration, and a scientific test, such as Dr. Gordon suggests, would probably revolutionise our regulations with regard to ventilation, and especially as they apply to buildings and apartments where large numbers of people congregate.

### Physical Characters and Morbid Proclivities.

A line of research which seems to offer some promise has been opened by Dr. F. C. Shrubsall, who has attempted to substantiate a correlation between racial types and disease. His observations are certainly suggestive. He finds that, speaking generally, blonde people are especially liable to acute rheumatism, heart disease, tonsillitis, and osteo arthritis; while nervous affections, pulmonary tuberculosis, and malignant disease show a predilection for individuals with dark complexions; while there are more doubtful indications of a correlation between tall stature and the former and short stature and the latter groups of disease. We shall look forward to the results of further and more extensive observations carried out on Dr. Shrubsall's lines.

### Surgery.

There is but little to say on the subject of the year's surgery. No striking developments have been manifest, although a great deal of sound and useful work has been done. Minor improvements in the technique of certain operations are almost all that can be definitely claimed. There are two tendencies, however, which may be referred to, viz., a growing inclination to operate early in acute appendicitis and other inflammatory processes within the abdomen, and, perhaps more important still, what appears to be a commencing reaction from the intemperate surgery of the last few years. To the thoughtful it has seemed certain that of late the ruddy triumphs of antiseptic surgery have led many surgeons to pass the limits of legitimate surgery; and to those who have observed the tendency a reaction will be very welcome.

### Accidental Hæmorrhage in Pregnancy.

At the meeting of the British Medical Association held at Oxford in July, a number of interesting discussions took place, but not one offered more material for reflection than that which followed Sir Arthur V. Macan's paper on the treatment of accidental hæmorrhage. Sir Arthur Macan advocates plugging the vagina for this complication of pregnancy, a procedure which has been attended in practice with eminent success. Now English teachers and text-books have emphatically and without reservation condemned this method, and the student who had the hardihood to recommend plugging the vagina in accidental hæmorrhage would almost certainly have been referred. We quote from a standard English text-book:—"Plugging the vagina is considered inadmissible in cases of accidental hæmorrhage, because concealed hæmorrhage might be going on behind the plug." The chief point is that plugging the vagina, as performed by Sir Arthur Macan and his Irish colleagues, has apparently been followed by a greater measure of success than has attended the treatment followed by the majority of English obstetricians. The fact illustrates one of the dangers of doctrine, and apart from its practical importance it bears a lesson which should never be forgotten by those whose privilege it is to teach and examine the rising generation of medical men.

### Medicine.

In the field of medicine, as in that of surgery, there is little that merits specific record. Considerable activity has been displayed in serum-therapeutics, and much benefit may be anticipated to result from investigations in this department of medicine. Pathology has been greatly to the front, and especially the pathology of blood, and the changes which occur in it in various conditions of ill-health. Apart from serum-therapy, medical treatment appears to be at a stand-still, and it is a growing complaint that physicians are becoming scarce, their rightful heritage being usurped by pathologists. Whether or not this is to be desired as a normal phase of evolution towards a better state we are not able to say, but the tendency is a remarkable one and deserves attention.

### Honours to Members of the Medical Profession.

In the past year there has been no prolific bestowal of titular honours upon members of the medical profession. The July list of birthday honours included the names of three medical men—Dr. Thomas Stevenson, scientific analyst to the Home Office, Dr. Constantine Holman, best known to medical men in connection with the British Medical Benevolent Fund and Epsom College, and Dr. Kendal Franks, C.B., who were knighted. An interesting feature of the honours list was the award of decorations in commemoration of the jubilee of the Crimean War. Among the recipients were Surgeon Thomas Ligertwood, who was made a Companion of the Bath in the Military Division. He served at Alma, Inkerman, and Sevastopol. Among the new Knight Commanders of the same Order were Deputy Surgeon-General James Howard Thornton, I.M.S., C.B., and Surgeon-General Edmond Townsend, A.M.S., C.B, C.M.G., Surgeon-General William James Fawcett, A.M.S., was made a Companion of the Bath in the Military Division, and Hon. Colonel John Edward Squire a Companion of the Bath in the Civil Division.

Mr. Bolton Glanvill Corney, M.R.C.S.Eng., Chief Medical Officer of the Colonial Government at Fiji, was appointed a Companion of the Imperial Service Order.

The November list of birthday honours contained the names of four medical men on whom was bestowed the honour of knighthood; the recipients were Mr. Charles H. Marriott, Surgeon to the Leicester Infirmary; Mr. Shirley F. Murphy, Medical Officer of Health to the County of London; Professor William Japp Sinclair, Professor of Obstetrics at the University of Manchester; and Major Allan Perry, R.A.M.C, principal Civil Medical Officer of Ceylon

The Illustrious and the Forgotten Dead.

Death has claimed his full portion from our ranks this year, and many a well-known leader has bowed his head and joined the great majority. But the honest worker, scarcely known outside the humble sphere of his daily round, and the great man whose name is known throughout the world, in death are equal, and equally merit a record on the tablets of the past. It is not for us to make invidious selections. Let them rest in peace, and equally in the honour and respect of those who remain.

